# Parents’ views on their own and their children’s well-being in the context of organised after-school activities in disadvantaged areas

**DOI:** 10.1186/s12889-026-29898-2

**Published:** 2026-10-14

**Authors:** Elisabeth Mangrio, Nour Zarzour, Margareta Rämgård

**Affiliations:** 1 Department of Care Science, Malmö University, Malmö, Sweden; 2 Research Centre Citizen Health, Malmö University, Malmö, Sweden; 3 Malmö Institute for Migration Studies, Malmö University, Malmö, Sweden

**Keywords:** After-school activities, All-Activity Houses, Children, Community-based intervention, Community-based participatory research, Multi-stage focus groups, Parents, Qualitative study, Well-being

## Abstract

**Background:**

In Sweden, a high proportion of the population have a migrant background. Many of them reside in socioeconomically disadvantaged areas and face challenges with integration, unemployment, and unstable housing conditions. In Malmö, Sweden, the municipality has established so-called All-Activity Houses, located in disadvantaged areas and offering after-school activities that are developed in close collaboration with the children themselves. There is a need to evaluate the effects of the All-Activity Houses and their activities on the children’s well-being. Therefore, using a community-based participatory research approach, the aim of the current study is to describe the parents’ views on their children’s and their own well-being, as well as the key social factors influencing it, in the context of participation in the All-Activity Houses.

**Methods:**

The method used was Multi-Stage Focus Groups, consisting of a series of focus group meetings conducted over an extended period during 2023–2024. Eighteen parents, with a background from nine different countries, chose to participate. The data were analysed with qualitative content analysis.

**Results:**

The results showed that social relations were strengthened by the activities in the All-Activity Houses, not only for the children but also for the whole family. The activities affected the well-being of the children in different ways, for example, by making them engage in physical activity. Besides helping the children to develop, the activities also increased their joy.

**Conclusions:**

This study demonstrated that the parents had a very positive attitude to the impact that the activities within the All-Activity Houses had on their children’s and their own well-being. The results also revealed that the parents wished for support in their parenting, and that further research is needed regarding parental support and its effect in similar communities.

## Background

In Sweden, a high proportion of the population have a migrant background and many reside in socioeconomically disadvantaged areas, facing challenges with integration, unemployment, and unstable housing conditions as well as economic challenges [1]. A disadvantaged, or vulnerable, area is defined by the Swedish Police Authority as a geographically delimited area characterised by low socioeconomic status, where criminal elements exert a significant influence on the local community [2]. Around 200,000 children in Sweden grow up in poverty, and four out of five of these children have parents with a migrant background; Malmö, Sweden’s third largest city, has among the highest rates of child poverty in the country [3]. Migrants on average also face challenges associated with social relations and integration into social networks in Sweden, including participation in civil society club activities as well as opportunities to meet people outside their immediate family and close friends, particularly those born and raised in Sweden [4–6]. Previous research in Sweden shows that migrants encounter barriers to integration related to, among other things, language learning and finding employment [4, 6, 7]. Similar challenges have also been identified in other contexts, such as Australia [8]. Obtaining stable and non-crowded housing conditions in Sweden can be a further challenge, especially without a work contract [4, 9], which is similar among migrants in, for example, the US [10]. Moreover, the level of trust among migrants in Sweden towards the people around them, and towards organisations and authorities in society, is quite low compared to that of the rest of the population [6]. Therefore, fostering trust in all interactions that migrants have with people in their community, as well as ensuring that organisations and authorities are perceived as trustworthy, is crucial. It is also important to consider the migrants’ opinions and thoughts on matters and processes in which they are involved.

A study of after-school activities among children in the US showed that children who regularly attended high-quality after-school programmes were reported by teachers to have higher academic performance, better work habits, and lower levels of aggression towards their peers [11]. An Australian study examined how socioeconomic status affects after-school activities and how it is linked to well-being [12]. They found that after-school activities differed by socioeconomic status: children from higher socioeconomic backgrounds engaged more in homework, sports, and reading, whereas those from lower socioeconomic backgrounds more frequently engaged in screen-based activities such as television, video games, and social media [12]. A key finding from this kind of research is that it is important for both children and parents to have access to social networks that can support them in everyday life. Social relationships and networks formed through school-based activities can contribute to the development of social capital among vulnerable families by connecting them to resources, information, and support. According to Robert Putnam [13], social capital is a valuable resource embedded in relationships and communities, just as economic capital is embedded in financial resources and human capital in skills and education. Putnam defines social capital in terms of norms of reciprocity, social networks, and trust that enable people to work together more effectively for mutual benefit.

To strengthen social capital among families with limited economic resources, Malmö municipality has established so-called All-Activity Houses (AAHs) in disadvantaged areas of the city. These areas are predominantly inhabited by people with a migration background, which means that a large proportion of children participating in the AAHs also have a migration background. The AAH is a programme provided at the school but offering after-school activities, primarily for children aged 6–16 years [14]. This programme thus provides children with free activities that are organised within the school setting. The activities may include sports, dance, music, cooking, and creative pursuits, as well as social gatherings in which the whole family can participate. One of the aims of the programme is to reduce inequalities among children in Sweden by expanding the programme nationally and building social capital in socioeconomically disadvantaged families. This aligns with Putnam’s theory of social capital, which suggests that people who participate in voluntary organisations, community activities, civic associations, and social networks tend to develop higher levels of cooperation and trust, thereby strengthening both individual well-being and societal functioning. Communities with high levels of social capital often experience better health, stronger democratic participation, lower crime rates, and improved collective problem-solving [13].

Putnam distinguishes between different types of social capital. *Bonding social capital* refers to strong ties among people who are similar, such as family members, close friends, or members of the same ethnic or religious group. Such groups can provide emotional solidarity, mutual support, and a sense of belonging. In contrast, *bridging social capital* connects people across different social groups, and these broader networks promote access to new information, opportunities, and social inclusion in a local community. Putnam argues that social capital in the US declined during the second half of the twentieth century. He observed decreasing participation in civic organisations, community groups, and other forms of collective engagement. This decline, he suggested, weakened social trust and community cohesion [13].

The activities at the AAHs in Malmö are developed in close collaboration with the children themselves, in order to strengthen social capital [3], and according to the staff at the AAHs, the schools involved have noted improvements in the children’s academic achievements. The three included AAHs opened in 2011, 2018, and 2022. Around 58% of the children aged 10–12 years attend an AAH, and slightly more girls than boys attend (51% girls, 49% boys). However, research on the AAHs was needed in order to generate evidence regarding the programme and its impact, while at the same time engaging the community in research on the cultural and social dimensions of their daily life. The research group conducting research on the AAHs, formed as community-based participatory research (CBPR) teams, has, since 2023, focused on both the children’s and the leaders’ perspectives (the study on leaders is not yet published) [14, 15]. This research addresses child well-being, which is understood in relation to children’s physical, emotional, and cognitive development [16]. In the overall programme, the concept of child well-being has been explored in greater depth through the children’s perspectives, drawing on Bronfenbrenner’s socioecological model. The children described well-being in terms of Enriching Leisure Time, Resourceful Places, Belonging to a Community, and a Society Built upon Welfare System and Rights [15]. These themes encompassed both structural aspects, such as school, healthcare, and human rights, and more personal dimensions, including close relations and places for social interaction in the children’s immediate environments. Thus, the children’s understanding of well-being reflects both individual and contextual dimensions, consistent with Bronfenbrenner’s ecological theory of human development [15].

A study among the children attending AAHs showed that they perceived the AAHs as strengthening social relationships in their local communities, which was important for their well-being. The children also highlighted how participation in the AAHs provided them with support to develop a sense of belonging through close relationships with both leaders and peers, as well as access to enriching leisure activities. Moreover, the findings demonstrated the importance of children’s ability to learn new skills and explore their identities through the social activities organised at the AAHs. The study indicated that promoting child well-being requires coordinated and planned participatory processes that support children’s empowerment [15].

Given their close relationship with the children, it is important to also consider the parents’ perspectives on how participation in social activities at the AAHs has affected their children’s well-being, as well as their own well-being as parents [14]. Children spend a significant amount of their time in school; therefore, the school environment is a key aspect for communities to consider in relation to children’s well-being. This is particularly important for children who face challenges growing up in disadvantaged areas [17], in view of the problems that these areas experience today in Sweden and other countries, where criminality and exposure to violence and drugs are increasing [18]. To the best of our knowledge, based on a review of the literature, only one study has examined parents’ experiences of being engaged in physical activities with their families in Sweden [19]. However, that study does not specifically address the focus of the current study, namely parents’ perspectives on well-being in the context of participation in AAHs. Research conducted in school-based settings has rarely involved parents’ perspectives, highlighting the need for further research in this area. Therefore, the aim of the current study, which forms part of a larger programme employing a CBPR approach, is to describe the parents’ views on their children’s and their own well-being, as well as the key social factors influencing it, in the context of participation in the AAHs.

## Methods

This study is part of a research programme called Kids in Action for Health Equity, which has a CBPR approach that has been presented before in a study protocol [14]. The programme is funded by the Swedish Research Council for Health, Working Life and Welfare (Reg. No. 2022 − 01043). The CBPR approach in this programme can be defined as a community-academic partnership between community members, parents, peer leaders, directors at the AAHs, and academic researchers who have gathered data in a co-creation process to build up a community capacity to improve health and social justice for members in the community. The research was conducted in the framework of the AAHs in three community schools, located on the outskirts of Malmö in southern Sweden in communities with a large number of migrants from all over the world and a high level of child poverty. The AAHs, directed by the municipality and the culture department in Malmö City, have employed peer leaders who serve as brokers. A broker is defined as someone working in disadvantaged communities and serving as a link, bridging people into the society [20]. The peer leaders are both younger leaders that themselves grew up within these areas and leaders that were educated within after-school activities, and some of them have an ethnic background similar to those of the children.

To be able to implement a CBPR approach it is necessary to reflect on your own role in research and on how to build trust in the research together with the community. The researchers in the present study have also taken part in the overall programme, initiating the research by attending the AAHs’ family activities and participating in them on different occasions. The first and last authors in this qualitative study have extensive experience of working as researchers as well as healthcare professionals (nurse and midwife) within similar communities. The first and last authors also have extensive experience in qualitative research. The first author, however, had not conducted focus group dialogues before, while the last author has considerable experience in conducting such dialogues.

### Design

The CBPR approach adopted in the overall programme also guided the present study, which employed qualitative inductive research based on several interviews and reflective dialogues with parents from diverse countries engaged in the programme. The study focused on parents that had children who participated in the activities at the AAHs. The role of the parents was to participate as research contributors by reflecting on their children’s and their own well-being in relation to the programme. The first author had a facilitating role, summing up each meeting and analysing the content of the discussions, using an interpreter when needed.

### Method

The method used was Multi-Stage Focus Groups (MSF), which involve a series of focus group meetings with focus groups interviews held over an extended period and are often used in participatory research [21]. The interview guide was developed specifically for the current study, but the questions were created within the broader research project that the current study is part of [14]. See Table 1.

**Table 1:** Interview guide

INTERVIEW GUIDE
What do you think about the activities for the children at the All-Activity House?
What effect do they have on the well-being of your child?
Have you noticed any differences in how the activities affect your child in relation to gender?

Paulo Freire [22] argues that critical reflection in knowledge creation can foster democratic participation among citizens in a community and contribute to social transformation. One purpose, besides focusing on the parents’ views on the children’s well-being, was to open up a reflective space for this kind of dialogues within a focus group setting. This approach allows dialogues and critical reflection to unfold in a cyclical or spiral manner, where each stage builds on the previous one and is informed by ongoing reflection and feedback in subsequent dialogues over time. In the first dialogues, the parents answered the three questions in the interview guide and discussed the meaning of well-being for their children, focusing on exploring and identifying aspects of children’s well-being related to their children’s participation in the AAHs. In subsequent meetings, these dialogues were followed by a deeper reflection on child well-being guided by the question ‘why’. This enabled the participants to deepen their understanding of well-being and explore the causes, consequences, and constraints affecting well-being in both the family and the community.

### Data collection

Parents were informed about the study through meetings related to their children’s participation in the AAH programme in Malmö. Approximately 50 parents were informed about the study and invited to participate. The information was provided during one face-to-face meeting at each of the three AAHs included in the study. The parents who expressed an interest in participating in the focus group interviews and dialogues then informed the AAH director, with whom they were acquainted. In the next step, information, both oral and written, was given to the parents by the first author. Eighteen parents – three fathers and 15 mothers – chose to participate; the participants could therefore be considered a self-selected sample [23]. Seven of the parents participated in two sessions per school and 11 of the parents only once. The parents represented nine different national backgrounds, including Kosovo, Syria, Albania, Iraq, Somalia, Palestine, Afghanistan, Morocco, and Kurdistan (see characteristics of the parents in Table 2). The focus group interviews were conducted on two to three occasions per AAH and, in total, seven focus group dialogues were held. They took place between June 2023 and September 2024 at the respective AAHs and were held in Swedish, with interpretation into Arabic and Somali. Each meeting lasted an average of 45 min. During the last meeting no new information emerged, indicating that saturation had been reached. The focus group meetings were audio recorded, and the recordings were securely stored.

**Table 2:** Characteristics of the parents who participated in the focus group meetings

Number of participants	Gender	Ethnic background	Repeat participation
18	3 men and 15 women	Kosovo (1) Iraq (5) Albania (2) Syria (4) Somalia (2) Palestine (1) Afghanistan (1) Morocco (1) Kurdistan (1)	7 (twice) 11 (once)

### Analysis

The first author analysed all the interview dialogues from the MSF and discussed them with the second and last authors, as well as with the research group. When all the focus group interviews were conducted, they were transcribed verbatim, and the data were coded by the first author. No field notes were taken. Then the last author read the coded material and gave feedback, in order to ensure credibility. Subsequently, the coded material was organised into clusters of codes with similar meanings, and through this coding process subcategories were formed. Main categories were then developed from subcategories with a common focus. The second author, who participated in the focus group meetings by facilitating the dialogues and helping to conduct the interviews, also reviewed the final version of the analysis and confirmed that it was consistent with the understanding developed during the data collection. This process can be seen as a form of face validity. The data were analysed with an inductive content analysis, following the method described by Burnard [24]. No software tool was used for the analysis. There was no member checking among the participants after the analysis was finished. Table 3 provides a few examples of the process of analysis from meaning units and codes to subcategories and main categories. The following steps were conducted:

1. Interviews were transcribed in Swedish and read.
2. Open coding was performed and used to create subcategories.
3. After clarifying the meaning of each subcategory, they were grouped into main categories.
4. The results were discussed by all authors and adjustments were made continuously until all data were used.

**Table 3:** Analysis schedule from meaning unit and code to subcategory and main category

Meaning unit	Code	Subcategory	Main Category
‘Before my daughter started participating in the activities, she had some difficulties speaking to and expressing herself with other children, but after she began attending the activities, her communication improved and she developed more relationships with the children around her’. (Focus group 2)	The daughter’s language and communication skills developed, and she built better relationships with the other children thanks to the activities.	Increased social connectedness between children	Social relationships, fostered by the AAHs, strengthen the local community
‘The importance of children being able to participate in activities, as it allows them to engage in physical activity’. (Focus group 1)	Activities promote physical activity.	Children become more physically active and use mobile phones and TV less	The group activities enhance the children’s overall well-being

## Results

The analysis described the parents’ views on their children’s and their own well-being, as well as the key social factors influencing well-being in the context of participation in the AAHs. It resulted in two main categories and seven subcategories. The first main category was Social relationships, fostered through the AAHs, strengthen the local community, with the following subcategories: *Increased social connectedness between children; Sense of community for the whole family; The important role of the leader;* and *A desire for support in parenting.* The second main category was *The group activities enhance the children’s overall well-being*, with the following subcategories: *The children become more physically active and use phones and watch TV less; The children develop self-esteem and independence through the activities;* and *The activities promote joy among the children*. See Table 4.

**Table 4:** Main categories and subcategories

Main categories	Subcategories
Social relationships, fostered through the AAHs, strengthen the local community	• *Increased social connectedness between children* • *Sense of community for the whole family* • *The important role of the leaders* • *A desire for support in parenting*
The group activities enhance the children’s overall well-being	• *The children become more physically active and use phones and watch TV less* • *The children develop self-esteem and independence through the activities* • *The activities promote joy among the children*

### Social relationships, fostered through the AAHs, strengthen the local community

The parents expressed how the activities helped their children have social relationships with other children and how they, as a family, were getting opportunities for social interaction with other families. The parents also highlighted the important role of the leaders for both the children and themselves. Lastly, they expressed a need for support as parents.

#### Increased social connectedness between children

The parents described how the activities led to their children engaging more with peers and how the children’s circle of friends was widened by getting to know other children through the activities. Since the children were often active on their phones when communicating with each other, the parents appreciated the fact that their children now more frequently met with other children in person. The parents also mentioned how their children developed their language skills and became more confident in expressing themselves after interacting with other children. One parent described how her children met with the other children after the activities and even visited other children’s homes. Another parent expressed it like this:

*‘Since I know the family through the All-Activity House*,* I feel safe when my daughter goes to her friend. But I’m very careful with families that I don’t know. But since we know this family through the All-Activity House*,* I just think this feels safe and positive*.’ (Focus group 2).

Yet another parent said that she was thankful for the social connections that her daughter developed during the activities. Before, it used to just be the mother and her daughter doing things together, but now the daughter could also do things on her own and spend time with friends.

#### Sense of community for the whole family

The parents reflected on the various excursions they had participated in as part of the AAH activities, which involved visits to other parts of the city. They expressed how much they appreciated these opportunities, especially since they were normally not able to travel far beyond the area where they lived, due to financial constraints. One parent said:

*‘My children need to get to know more places around here*,* so that they can tell other kids about how they went here and there*,* for example: “We went to Tosselilla Summer Park”. That feels good for me as a parent*.’ (Focus group 1).

Furthermore, the parents described how they liked to do activities together as a family but also to meet with other families. Some parents discussed the importance of such social interaction for their learning of Swedish. Some of the mothers talked about going to dance classes organised by the AAH, and how good it felt to just be among other mothers and relax from the workload at home. Other parents commented on the ‘family parties’ arranged by the AAHs and expressed how much they appreciated being able do fun activities with their children, meet other parents, exchange contact information, and then try to arrange future gatherings with these families.

#### The important role of the leaders

The parents also reflected on how safe they felt with the leaders of the AAH and described how the peer leaders talked to and listened to their children. The way the peer leaders interacted with and affirmed the children was highly appreciated. One parent talked about how one of the leaders had called her:

*‘The leader called me and told me about how well my daughter had done in the singing and dancing activities*,* and it felt so good. I was so proud of my daughter.’* (Focus group 3).

Several other parents also discussed how important it was for them to get feedback on how their children were doing at the AAH and how much they valued this interaction with the peer leaders. Another parent described how amazing the peer leaders were, saying that in the school the teachers taught the children knowledge, but at the AAH the leaders taught the children friendship and social values. They claimed that their children really felt safe at the AAH and that the leaders genuinely invested in the children, as demonstrated by the children’s eagerness to return.

#### A desire for support in parenting

The parents mentioned that it would be great if they could also attend some parental support groups with other parents in their own community, especially for those who spoke little Swedish, and where they could learn from each other. This support was considered particularly needed as the children were growing and getting older. One mother described a course she had attended, organised by a local women’s association, in which they learnt about the support available from the social services and how they therefore did not need to fear them. Another mother said how she would like to get more information from the social services and the police, since many parents saw them as a threat. She argued like this:

*‘When the social services get a report of worry regarding a family*,* it’s very stressful for the family involved and it can make them feel threatened. It would be good to understand the steps that a “worry report” entails and to understand how it works*,* to make us able to deal with it and be less worried*.’ (Focus group 5).

Yet another mother said that if she was waiting for her daughter and she did not come home, it would be good to call the police, but that she would not do so because she believed that the police would probably report her to the social services. This, she argued, led to a reluctance to seek support and help that could actually be needed. Several parents expressed a need for more knowledge regarding parenting and available support services in Sweden.

### The group activities enhance the children’s overall well-being

The parents expressed how the activities affected the children’s well-being in different ways, such as making them engage in physical activity instead of spending their time in front of the TV or scrolling on their mobile phones. The parents sensed that the children developed in different ways, both by learning new skills, such as baking, and by developing their self-esteem and confidence. The parents also expressed how happy the activities made the children, saying that they could see a change in their children’s mood after participation.

#### The children become more physically active and use phones and watch TV less

Several of the parents mentioned how good the activities at the AAH were, since they made the children engage in physical activity, such as playing football or dancing. This was good because of the health benefits but also because it decreased the children’s use of phones or computers. One mother said the following:

*‘For me as a mother it’s very important that my children go and play and do physical activities*,* which reduces their otherwise excessive use of phones and computer*s.’ (Focus group 1).

Other parents commented that one activity they missed was swimming. As many children did not know how to swim, the parents felt that including swimming among the activities offered by the AAH would be beneficial. One mother had experienced her daughter nearly drowning, and therefore considered swimming a particularly important activity to offer the children.

#### The children develop self-esteem and independence through the activities

Some parents talked about how the activities had contributed to their children’s development, strengthening their self-esteem and independence. One parent noted that her daughter’s confidence in singing had increased since participating in the programme. Another parent said that her son was shy before starting the activities, but that now he had become more confident. Other parents talked about how activities such as dancing had made their children feel freer and able to express themselves. One father said that he could see how his son’s thinking had developed, reflecting on this as follows:

*‘Before my son started the activities*,* he did not feel that good and he did not talk and ask as much as now after the activities. It has developed his thoughts and analytical skills and he asks more questions*,* which is a good development.’* (Focus group 4).

Other parents talked about how the children learnt new skills, such as baking, and how they showed their skills at home. Seeing all that their children had learnt made the parents happy.

#### The activities promote joy among the children

Parents’ discussions also concerned how the activities made the children happier and how they could notice a difference in the children after they had attended the activities. They could also sense joy within their children on the days when they had activities, noting how much they looked forward to them. One parent talked about joy being important for children’s well-being. Another parent told the other parents about the children going to restaurants as part of the activities and how happy her daughter was about this. One mother described her daughter’s appreciation of the AAH activities:

*‘She really likes this. She gets some break from school and homework and the stress to do well in school*,* and she’s always happy when she comes home*,* telling me about all the things that she’s done*.’ (Focus group 7).

One parent lived far away from the AAH, but their daughter still stayed there after school before taking the bus home. The parent remarked that if their daughter had not enjoyed the activities, she would never have taken the time and made the effort to attend them.

## Discussion

In this study, the parents had the opportunity to reflect on the role of the AAH in their children’s and their own well-being, as well as on the factors that caused this well-being from their point of view. The results revealed that the AAHs in disadvantaged areas were important not only for the children themselves but also for their families and the community. Although the questions in the interview guide focused on the children and their well-being, the parents started to reflect more deeply and describe situations in which they, as families, felt included within the AAH community. This is of particular importance, as many people residing in these communities may feel excluded from wider society and may therefore be in need of supportive social relationships [4, 6–8]. The AAHs seemed to be able to meet such needs.

Furthermore, the results showed that the parents had a very positive attitude to the impact that the activities within the AAH had on their children’s well-being. In addition to highlighting relational benefits, such as increased social connections both among the children and for the whole family, the results emphasised the crucial role of the peer leaders. Parents described the leaders as important links between children and families within the community, which could be related to the fact that some of the leaders grew up in these areas and spoke the same language as the children. From a social capital perspective, the leaders at the AAHs can be understood as brokers who facilitate bridging social capital between different groups participating in the activities. The leaders’ role within the AAHs is vital in many respects. By supporting children in these socioeconomically disadvantaged areas they can indirectly prevent involvement in criminal activity, as a lack of role models around the child constitutes a risk factor for such activity [25]. The importance of leaders is also highlighted in other studies focusing on disadvantaged areas and work with activities for children [26, 27]. Peer leaders not only have a good effect on the children, but their work could also benefit the leaders themselves [27]. A recently published study of children participating in AAHs further demonstrated that the leaders play a key role in children’s well-being, with children valuing access to a trusted adult outside their family and school contexts [15].

In the present study, it also emerged that the parents wished for support in their parenting, and that the shared reflection within the group seemed to be important to them, despite cultural differences, as also reported in an earlier study [4].

Moreover, the results of the present study demonstrated how the activities organised by the AAHs benefited the children’s well-being by making them physically active, developing them both cognitively and physically, and increasing their joy. These are all important aspects to consider, not least because today the mental well-being of children is increasingly challenged [28], but also because children in socioeconomically disadvantaged communities may be more likely to engage in social media use and video gaming than to participate in physical activities [12].

A recent study from Sweden showed that migrant parents struggle to raise children in areas with a high crime rate. They express concerns that deviant behaviour may become normalised in these areas and are worried that their own children will adapt to the local environment and develop risk behaviours [29]. The results of that study demonstrated that social capital is important for families in these communities and that the AAH could be a valuable meeting point, not only for the children, but also for the families to connect with others they can trust and rely on. An earlier study done in the US showed that children who attended a high-quality after-school programme on a regular basis showed less aggression towards their peers [11]. According to Putnam [13], community social capital for local capacity building can be created through bonding and bridging social networks. He argues that strong social networks, trust, and civic engagement are essential resources for individuals and societies, as social capital facilitates cooperation, strengthens communities, and contributes to social well-being among the people of the community [13]. The findings of this study suggest that the AAHs’ activities strengthen bonding social capital within the children’s own family, while also fostering bridging social capital between families with different cultural values, which may be particularly important. The AAH could, furthermore, be understood as an environment that connects families in the community to the local municipality, thereby facilitating linking social capital. The concept of linking social capital has been described and discussed by Simon Szreter and Michael Woolcock, and refers to connections between individuals or groups and institutions or authorities, such as schools [30]. This may lead to capacity building and trust in the local communities in the city of Malmö, where AAHs are implemented.

It is, moreover, important to further develop the social cohesion created within the AAHs not only among the children but also among their families. The results of the current study showed that the parents appreciated the social interaction between different families at the AAH and felt that it benefited them. This is an important aspect, as earlier research indicates that migrants may lack social relations, which may in turn increase mental ill-health [4, 5]. Participating in community activities can also help migrant parents integrate and improve their Swedish language skills, as suggested by previous research [4]. Furthermore, the existence of child poverty, especially in disadvantaged areas [31, 32], should be considered. This may, for example, make it difficult to go on family trips, and activities arranged by the AAHs for the whole family could therefore serve as a substitute for trips that more well-off families can afford.

One question in the interview guide focused on gender and its impact on well-being. When this question was raised in the focus group meetings, few of the parents perceived any differences in how gender affected the well-being of their children in relation to the activities. Therefore, nothing in the results was related to this matter. Hopefully, studies in the near future could provide further insight into this question.

Further studies of programmes such as AAHs are needed to quantitatively investigate the effect that such programmes may have on children’s well-being, using social capital as an outcome measure.

## Limitations

All research studies have limitations. In this study, migrant parents from nine different countries participated and not all of them spoke Swedish, but we had access to Somali and Arabic interpreters. There is always a risk that more parents who are doing well and have a more favourable situation socially participate. This risk could be related to the fact that only parents who attended the information meetings about the interview study were able to receive information about the study and decide whether they wanted to participate. It could be assumed that parents who attend such meetings are, in general, more integrated into society than those who do not. We do not have sociodemographic information on the parents who did not attend the information meetings, which could be considered a limitation of the study. However, we had a diverse range of parents, with varying Swedish skills: some spoke a little, some more fluently, and some not at all.

Another limitation may stem from the fact that 11 of the 18 parents that participated in the study attended only one session. In order to mitigate this limitation, we started each session with a summary of the topics covered in the previous session.

We used interpreters and are aware that a power imbalance may have occurred, as interpretation may be affected by how participants and interpreters perceive each other [33]. But we had the same Arabic interpreter for all the sessions, and she engaged with the families before each session, ensuring that they would attend and that they felt safe with her. She thus functioned as a cultural broker [34]. We also employed a Somali interpreter who was well regarded both by the parents and by the researchers. This could have contributed to limiting the power imbalance.

The focus groups were conducted at three different AAHs and with different parents, but the interview responses and the discussions were similar in all the groups, which strengthens the credibility of the results. The analysis of the results was validated by discussions within the research group, whose members had previously conducted focus groups with leaders and children at the AAHs. The similarities between the findings provided further support for the analysis. Dependability was ensured [35] by adhering closely to the analytical process described by Burnard [24]. It would have been beneficial to conduct member checking with the participating parents, but this was not possible for logistical reasons. Finally, another limitation was the fact that the interview guide was not tested prior to the focus group meetings, although this could have assessed the validity of the tool. However, the interview questions seemed easy to understand for the parents.

## Conclusions

The results of this study showed that the parents perceived the activities arranged by the AAHs as having a very positive impact on their children’s well-being. Above all, this impact had relational aspects, such as promoting social connectedness both for the children and for the whole family. Moreover, the results highlighted the important role of the peer leaders in supporting the children’s well-being. The results also revealed that the parents wished for support in their own parenting, and further research is needed on parental support and its effect in similar communities.

## Data Availability

The datasets generated or analysed during the current study are available from the corresponding author on reasonable request.

## Abbreviations

AAH: All-Activity House
CBPR: Community-Based Participatory Research
MSF: Multi-Stage Focus Groups
